# Cold-water gut isolate from threespine stickleback (*Gasterosteus aculeatus*) reveals polypropylene surface oxidation and co-culture inhibition

**DOI:** 10.1128/msystems.00599-26

**Published:** 2026-09-30

**Authors:** Sarah M. Pasqualetti, Jolie Atwood, Abiodun Aderibigbe, Edward Russell, Kayleigh O'Keefe, Victoria Rosario, Kelly Ireland, Kenneth Sparks, Koral Campbell, Ruth Y. Isenberg, Ryan Lucas, Kathryn Milligan-McClellan

**Affiliations:** 1 Department of Molecular and Cell Biology, University of Connecticut 7712, Storrs, Connecticut, USA; 2 Department of Chemistry, University of Connecticut 7712, Storrs, Connecticut, USA; 3 Department of Biology, University of Alaska Anchorage 3291, Anchorage, Alaska, USA; 4 Department of Social and Critical Inquiry, University of Connecticut 7712, Storrs, Connecticut, USA; University of California San Diego, La Jolla, California, USA

**Keywords:** plastic biodegradation, polypropylene, polyethylene terephthalate, threespine stickleback, cold-water bacteria, bioremediation, co-culture dynamics

## Abstract

Polyethylene terephthalate (PET) and polypropylene (PP), two of the most widely produced plastics in the United States, persist in diverse environments worldwide and may persist longer in cold-water environments where plastic-degrading microbial taxa have been poorly characterized. Understanding how gut microbes interact and contribute to plastic degradation is essential for developing potential microbiome-based bioremediation strategies. We isolated 184 microbes from wild Alaskan threespine stickleback (*Gasterosteus aculeatus*) guts across six bodies of water and screened them for their plastic-degrading potential using lipase/esterase assays and biofilm formation on PET and PP. We discovered that while some members of the stickleback gut microbiota have high lipase, esterase, and biofilm activity, that activity is enhanced or suppressed by other microbes. Isolates with the highest plastic-degrading potential were incubated in minimal media with PET or PP as the primary carbon source to determine whether plastic degradation occurs. While no detectable changes were observed on PET, surface analysis identified a *Pseudomonas trivialis* strain that exhibited evidence of PP surface oxidation in monoculture; however, this activity was suppressed in the presence of another gut isolate, *Pseudomonas germanica*. These results demonstrate that microbes associated with the gut microbiome of a cold-water fish possess plastic-degrading potential and provide insights into how microbial interactions can inhibit bioremediation of plastic pollution in cold-water environments.

**IMPORTANCE:**

Plastic pollution persists in cold, freshwater environments and presents a significant challenge to the ecosystem, where low temperatures slow abiotic degradation, and microbial contributions remain poorly understood and understudied. Here, we isolated 184 microbes from adult Alaskan threespine stickleback guts across six bodies of water and screened for plastic-degradation potential using plate-based assays under both monoculture and co-culture conditions. We identified a stickleback gut-associated microbe that can oxidize polypropylene and identified a second microbe, isolated from the same environment, that can significantly suppress polymer oxidation when in co-culture. Together, these findings demonstrate that microbial interactions influence plastic-degradation abilities and support efforts to identify ecologically relevant microbes or enzymes for bioremediation efforts.

## INTRODUCTION

Plastic waste, such as polyethylene terephthalate (PET) and polypropylene (PP), is ubiquitous in the environment and degrades slowly (1–3), resulting in microfragments that threaten biodiversity and ecosystem function. This highlights the need for more effective waste management, particularly in aquatic ecosystems where its impact is most significant (4–7).

In colder climates, both abiotic and biotic degradation are slowed; thus, plastics may persist longer in these environments (8). Microbial degradation has emerged as a promising approach toward mitigating plastic pollution, yet most characterized plastic-degrading microbes come from soil or temperate marine settings (9–14), leaving cold freshwater systems underexplored. Several terrestrial bacterial species have been shown to degrade plastic, such as *Ideonella sakaiensis* 201-F6, which uses PET as a carbon source and produces the enzymes required for degradation (15), as well as multiple *Bacillus* and *Pseudomonas* species (12, 16–19).

Moreover, many natural degradation processes involve interactions among microbial species. Co-culturing microbes has been shown to enhance plastic degradation (12, 19, 20), evidenced by enhanced enzyme activity and detectable surface modifications to plastic samples. These synergistic effects likely stem from complementary metabolic processes such as cross-feeding or genetic co-regulation associated with degradation pathways (21, 22). However, antagonism between species can suppress degradation potential. Understanding both cooperative and inhibitory dynamics is therefore essential in identifying potential plastic-degrading microorganisms in underexplored environments.

Given the global scale of plastic pollution, there is a need for accessible and relatively cheap screening methods to assess microbial plastic-degradation potential. Similar large-scale screens have been conducted in other studies aiming to identify plastic-degrading organisms (23, 24) but not in cold freshwater ecosystems, highlighting an important gap in knowledge.

Microbial biofilms commonly form on plastic surfaces and facilitate prolonged interactions between the microbial community and the plastic surface (25). Biofilms are known to facilitate plastic degradation by concentrating plastic-degrading enzymes near the plastic surface (26, 27). Next, the microbial community secretes extracellular enzymes that interact with polymer surfaces and contribute to degradation processes. Lipases and esterases are commonly used as indicators of plastic degradation (12, 28) because long-chain hydrocarbons found in polymers such as PP share structural similarities with natural lipid substrates (29), while ester-containing plastics such as PET are susceptible to enzymatic hydrolysis (29–34). Ultimately, complete degradation and mineralization can result in the release of carbon dioxide and water (15).

To evaluate the ecological relevance of plastic degradation in cold-water environments, we explored host-associated microbes in the threespine stickleback fish (*Gasterosteus aculeatus*), a cold-water species found across the Northern Hemisphere (35). In stickleback, gut microbial communities include *Pseudomonas* and *Bacillus* species (36). Alaskan lakes are contaminated with plastic (37), which could contribute to the presence of potential plastic-degrading enzymes. Because fish interact with aquatic environments through feeding and environmental exposure, the gut microbiota may serve as a reservoir of microbial functions shaped by local environmental conditions (38, 39). Furthermore, gut microbes from other species have been associated with plastic degradation (40–42). Therefore, using microbes from wild-caught Alaskan stickleback could provide ecologically relevant bacterial strains that could be used for bioremediation in cold-water environments.

Here, we asked whether gut-associated microbes isolated from wild Alaskan threespine stickleback have plastic-degrading capabilities and whether microbial interactions modulate surface modification. We isolated 184 microbes from wild Alaskan stickleback across six bodies of water and screened them for plastic-degrading potential by determining lipase and esterase activities and biofilm formation on PET and PP films. We quantified PP and PET surface modifications using attenuated total reflectance Fourier transform infrared spectroscopy (ATR-FTIR) and X-ray photoelectron spectroscopy (XPS) and characterized plastic surface modifications using scanning electron microscopy (SEM). We also tested co-cultures to evaluate whether microbial interactions could enhance or inhibit plastic surface modification. We hypothesized that microbes with positive enzyme activity, biofilm formation, and detectable surface modifications on plastic films would have the greatest plastic-degrading potential and that co-cultures would modulate these effects through synergistic or inhibitory interactions.

## MATERIALS AND METHODS

### Microbe isolation

Microbes were isolated from healthy adult threespine stickleback guts from four freshwater and two anadromous populations in Southcentral Alaska (Table 1) under University of Alaska Anchorage IACUC protocol 907288, Alaska Department of Fish and Game (ADF&G) permit number P16-003, University of Connecticut IACUC protocol A21-021, and ADF&G permit number P-21-012. Fish were wild caught between 2016 and 2022 using minnow traps and processed to isolate individual microbes as described in Milligan-McClellan et al. (36). Each microbe was given a unique Kat Milligan-McClellan (KMM) number.

**TABLE 1:** Characteristics of bodies of water from which fish were collected and microbes were isolated^*a*^

Lake name	Collection coordinates	Elevation (m)	Area (ha)	Maximum depth (m)	Human activity^*b*^
Bear Paw Lake (43)	61.613948, −149.752637	82	18.2	5.2	Low
Big Lake (43)	61.54787, −149.85357	43	1,009.7	27.1	High
Cheney Lake (43, 44)	61.20490, −149.75940	61	9.9	4.9	High
Mud Lake (43)	61.5625, −148.9486	791	16.2	2.4	Low
Rabbit Slough (45)	61.53666, −149.2533	13	NA	NA	Moderate
Westchester Lagoon (43, 45, 46)	61.20622, −149.92423	4	33	4.0	High

^*a*^ NA, data not available. Rabbit Slough is a shallow creek/slough system; therefore, area and maximum depth measurements are not available.

^*b*^ Human activity was determined based on recreational activity, housing density, and location.

### Microbe identification

Microbes were identified by amplification and Sanger sequencing of the 16S or 18S ribosomal RNA gene using the 27F and 1492R universal primers (47) for 16S or the Euk1A-F and 564R universal primers (48) for 18S (Genewiz, New Jersey). Aligned consensus sequences were compared to NCBI’s core nucleotide database using the Basic Local Alignment Search Tool for Nucleotides (49).

### Culture conditions

Microbes were streaked for isolation onto LB agar from original freezer stocks, incubated with shaking to mid-log phase at 25°C in LB, and diluted to an OD_600_ of 0.5 for lipase, esterase, and biofilm assays. Strains 9.1 (*Bacillus thuringiensis* C15) and 9.2 (*Pseudomonas* sp. B10) are part of a consortium reported to degrade PET (12) and were used as positive controls. For co-culture experiments, six representative isolates were selected based on results from the initial screening assays, including lipase/esterase activity, biofilm formation, and microbe identification. Co-culture pairs are outlined in Table S3.

### Lipase assay

#### Qualitative plate screening

Lipase activity of individual isolates was first screened on rhodamine B agar plates. The agar was prepared following the Alken Murray Corporation (50) with modifications. Lipids were emulsified in a Conair Waring Laboratory Blender (22,000 rpm, 5 min). Plate inoculation was performed as described by Roberts et al. (12) with modifications. A 5 µL aliquot of diluted culture was spotted onto triplicate rhodamine B agar plates and incubated at 25°C. Fluorescent orange halos surrounding the colonies under UV illumination (302 nm) indicated enzymatic hydrolysis of lipids, which was recorded at 24 and 48 h (51).

#### Quantitative co-culture assay

To compare lipase activity between monocultures and co-cultures, a quantitative liquid lipase assay was adapted from Zottig et al. (52). Liquid media were adapted from the Alken Murray Corporation (50), containing 0.9% (wt/vol) nutrient broth, 0.25% (wt/vol) yeast extract, and 0.02% (wt/vol) rhodamine B. Assays were conducted in black, clear-bottom 96-well plates (Corning, cat. 3603) containing 190 µL of medium. Wells were inoculated with 10 µL of a single isolate (monoculture) or 5 µL of each of two isolates (co-cultures). Culture plates were incubated without shaking at 25°C for 48 h.

#### Fluorescence measurement and statistical analysis

All lipase assays were performed using a minimum of three technical replicates for each isolate or isolate combination. Fluorescence was measured as relative fluorescence units (RFU) at 0, 24, and 48 h on a SpectraMax i3x Plate Reader (350 nm excitation and 580 nm emission) (52). Prior to each measurement, plates were shaken for 5 s at medium speed. The effect of co-culturing was quantified by calculating the log_2_ fold change in fluorescence relative to the corresponding monocultures. Fold change data were evaluated using both one-sample *t*-tests and Wilcoxon signed-rank tests relative to a null expectation of zero.

### Esterase assay

Tributyrin agar (1% and 2%) was prepared as described by Ramnath et al. (53), with modifications: 20 µL of diluted culture was added to 6 mm punch-outs created in agar plates. Esterase screening assays were performed using three technical replicates for each condition. For co-culture assays, wells received either 20 µL of a single strain of isolate (monoculture) or 10 µL of each strain of two isolates (total 20 µL). Plates were incubated at 25°C for 48 h. Esterase activity was inferred from clearing zones produced by tributyrin hydrolysis. The diameter of each clearing zone (edge-to-edge, inclusive of the punch) was measured at 24 and 48 h. Total hydrolysis area (*A*_Hydrolysis_) was calculated using the following equation:


$$A_{Hydrolysis}=\pi \left[{\left(\frac{d_{clear}}{2}\right)}^{2}-{\left(\frac{d_{punch}}{2}\right)}^{2}\right]$$

where *d*_clear_ is the clearing zone diameter and *d*_punch_ = 6 mm.

### Statistical analysis

All statistical analysis was performed in Prism 10. Monoculture samples were compared to positive control 9.1 *Bacillus thuringiensis* using one-way ANOVA. For co-culture assays, log_2_ fold changes in hydrolysis area were computed relative to the corresponding monoculture(s), and one-sample *t*-tests and Wilcoxon signed-rank tests were used to assess whether log_2_ fold changes differed significantly from zero.

### Biofilm formation assay

#### Preparation of surfaces and assay setup

PP (Millipore Sigma, cat. GF99473552) and PET (Millipore Sigma, cat. GF31588229) films were cut into 1 × 1 cm squares and sterilized in 70% ethanol. The assay was performed in 48-well polystyrene (PS) culture plates (Fisher Scientific, cat. 08-772-52) containing LB. The experimental treatments consisted of wells containing (i) a vertically oriented PET film, (ii) a vertically oriented PP film, or (iii) no film (control). Microbes were either added to the culture plate directly from an agar plate (initial screen) or grown in an overnight culture with shaking at 25°C (quantified and reported assay) at a final OD_600_ of 0.002. Plates were incubated at 25°C without shaking for 68 h.

#### Biofilm staining and quantification

Biofilms formed on the plastic films and on the surfaces of the PS wells were quantified separately. Plastic films were transferred to a new 48-well plate for analysis. Both the original plate (containing well-adhered biofilms) and the new plate (containing film-adhered biofilms) were processed for staining as described in Chen et al. (54). Crystal violet was aspirated from the wells and films, rinsed in deionized water, and dried at 30°C overnight. Crystal violet was extracted with 30% (vol/vol) glacial acetic acid (Sigma, cat. A6283), and the absorbance was measured at 590 nm.

#### Data normalization

The relative biofilm formation (RBF) for each replicate was calculated using the following equation:


$$RBF=\frac{A_{590}(Sample)}{A_{590}(Blank)}$$

Relative biofilm formation for each condition was normalized (RBF_Normalized_) using the following equation:


$$RBF_{Normalized}=\frac{RBF_{Sample}-RBF_{Control}}{Total\;Surface\;Area\;({cm}^{2})}$$

To compare biofilm growth on different surfaces, two-way ANOVA with Tukey’s multiple comparison test was used to analyze differences between the PET, PP, and no-plastic control conditions (55). To assess co-culture interactions, one-way ANOVA with Tukey’s multiple comparison test was used to compare biofilm formation in co-cultures to their respective monocultures (55). Additionally, the log_2_ fold change in relative biofilm formation for co-cultures was calculated, and one-sample *t*-tests and Wilcoxon signed-rank tests were used to determine if these changes were significantly different from zero. All statistical analyses were conducted in Prism 10. For the representative subset of isolates highlighted in Table 2, monoculture biofilm assays were independently repeated using three biological replicates, each of which had three technical replicates. Co-culture biofilm assays were performed using three technical replicates per co-culture combination.

**TABLE 2:** Identifications of the representative subset of microbes highlighted in the results^*a*^

Isolate	Species	Lipase activity	Esterase activity
KMM 191	*Pseudomonas germanica*	+	+
KMM 193	*Pseudomonas fragi*	+	+
KMM 195	*Pseudomonas trivialis*	+	+
KMM 200	*Erwinia persicina*	−	−
KMM 202	*Pantoea agglomerans*	−	+
KMM 230	*Pseudomonas germanica*	+	+

^*a*^ Lipase and esterase activity results were based on plate-based assays.

### Biofilm assays, fourier transform infrared spectroscopy, and X-ray photoelectron spectroscopy

#### Culture conditions

Minimal salt medium (MSM) was modified from Augusta et al. (56) and contained 0.1% (wt/vol) NH_4_NO_3_, 0.07% (wt/vol) KH_2_PO_4_, 0.07% (wt/vol) K_2_HPO_4_, 0.07% (wt/vol) MgSO_4_·7H_2_0, 0.5% (vol/vol) Wolfe’s trace vitamins, and 0.5% (vol/vol) trace mineral solutions. Liquid cultures were grown to mid-log phase in LB, pelleted at 8,000 × *g* for 10 min, and resuspended in MSM and inoculated into a glass tube containing 4 mL MSM and 1 × 1 cm PP or PET films to a final OD of 0.1. Monocultures contained a single strain, while co-cultures contained equal volumes of each strain. Tubes were incubated at 25°C for 8 weeks under static conditions. Fresh media were added to each tube at 4 weeks. Uninoculated control tubes containing MSM and plastic films were incubated under identical conditions for the full 8-week period. At the end of the incubation period, the plastic films were removed and divided in half for use in biofilm, FTIR, and XPS.

#### ATR-FTIR

Plastic films were treated with enzymatic lysis buffer containing 20 mg/mL lysozyme overnight at 37°C to remove the cells and biofilm, followed by three rinses with ddH_2_O water and drying at 30°C.

ATR-FTIR spectra were collected according to Roberts et al. and Kyaw et al. (12, 18) with modifications. Spectra were collected on a Nicolet iS20 FTIR spectrometer using OMNIC software (Thermo Fisher Scientific) over a range of 4,000–400 cm^−1^ at a resolution of 8 cm^−1^ and 16 scans per collection. Analyses were performed using two biological replicates per treatment. Within each biological replicate, three plastic films were analyzed as technical replicates. Each plastic film was measured in triplicate, resulting in nine measurements per biological replicate. Averages were compared both to uninoculated controls and an untreated plastic film that had not undergone incubation. To quantify the formation of carbonyl groups, the carbonyl index of PP was calculated using the specified area under band (SAUB) method (57):


$${Carbonyl\;Index}_{PP}=\frac{Area\;under\;band\;(1850-1650\;{cm}^{-1})}{Area\;under\;band\;(1500-1420\;{cm}^{-1})}$$

The carbonyl index of the PET was calculated according to Roberts et al. (12):


$${Carbonyl\;Index}_{PET}=\frac{Absorbance\;(1713\;{cm}^{-1})}{Absorbance\;(1410\;{cm}^{-1})}$$

Treated plastic films were compared to a negative control using one-way ANOVA for the monocultures and two-way ANOVA followed by Tukey’s multiple comparison test for the co-culture assays.

#### XPS

XPS was performed according to Yang et al. (42) with modifications. XPS was recorded on a Thermo Fisher Scientific K-Alpha spectrometer with a monochromatic Al Kα source (hv = 1,486.6 eV). Samples were mounted using double-sided carbon tape. The X-ray spot size was 400 µm; the takeoff angle was 90°; and the analyzer chamber pressure was maintained below 5.0 × 10^−8^ mbar. XPS analyses were performed using two biological replicates per treatment. For each biological replicate, three plastic films were analyzed as technical replicates, and three XPS scans were collected from each film at separate surface locations. Surface charging was mitigated using an electron flood gun. Survey scans were acquired at 200 eV pass energy with a 1.0 eV step size, and core scans were acquired at 50 eV with a 0.1 eV step size. Dwell time was 50 ms per point. Spectra were analyzed and peak fitted in CasaXPS according to Beamson and Briggs (58) with charge correction to C 1s photoelectron line at 285 eV.

Peak area percentages between the monoculture, co-culture, and uninoculated control were compared using two-way ANOVA followed by Tukey’s multiple comparison test (59) in Prism 10. Differences in overall peak distribution were assessed by pairwise permutational multivariate analysis of variance (PERMANOVA) in R (60) (v4.5.1) using the Vegan package (61) (v2.7-1), with Bray-Curtis dissimilarities and 9,999 permutations.

#### SEM

The same plastic films analyzed by FTIR were examined for surface modifications using SEM at the University of Connecticut’s Bioscience Electron Microscopy Laboratory CORE Facility. Sample preparation and imaging were adapted from Jeon et al. (17). Samples were mounted vertically onto double-sided carbon tape on a variable-tilt specimen holder and sputter-coated with a 5 nm layer of Au-Pd (80:20) using a Safematic CCU-010 Sputter Coater. Imaging was performed at 90° with an FEI Nova NanoSEM 450 equipped with an Everhart-Thornley Detector under high-vacuum conditions.

## RESULTS

### Identification of stickleback gut isolates

Given the large data set, a subset of isolates will be highlighted in the results for lipase, esterase, and biofilm forming abilities in monoculture and co-culture (Table 2). The full results of all 184 isolates from six stickleback populations are in Table S1.

### Hydrolytic enzyme activity varies across stickleback gut isolates

Lipase and esterase activities varied widely across the stickleback gut isolates. Of the 184 isolates, 74 were positive for lipase activity, with activity varying among strains within the same genera (Table S1).

Esterase activity was more prevalent, with 138 of 184 isolates showing hydrolysis (Table S1) on tributyrin agar. Overall activity patterns were consistent across both tributyrin concentrations; therefore, results are reported for hydrolysis on 1% tributyrin agar. Twenty-one esterase-positive microbes had at least 1.6 times higher esterase activity (*P* < 0.05) than positive control 9.1 (104.4 ± 0.0 mm^2^), ranging up to 173.3 mm^2^ (Table S2). Out of these 21 isolates, all were *Aeromonas* or *Pseudomonas* species except *Chromobacterium aquaticum* (KMM 293*,* 173.3 ± 25.2 mm^2^). KMM 191 (*P. germanica*) and KMM 195 (*Pseudomonas trivialis*) were among the top-performing isolates (Table S2).

**Fig 1:**
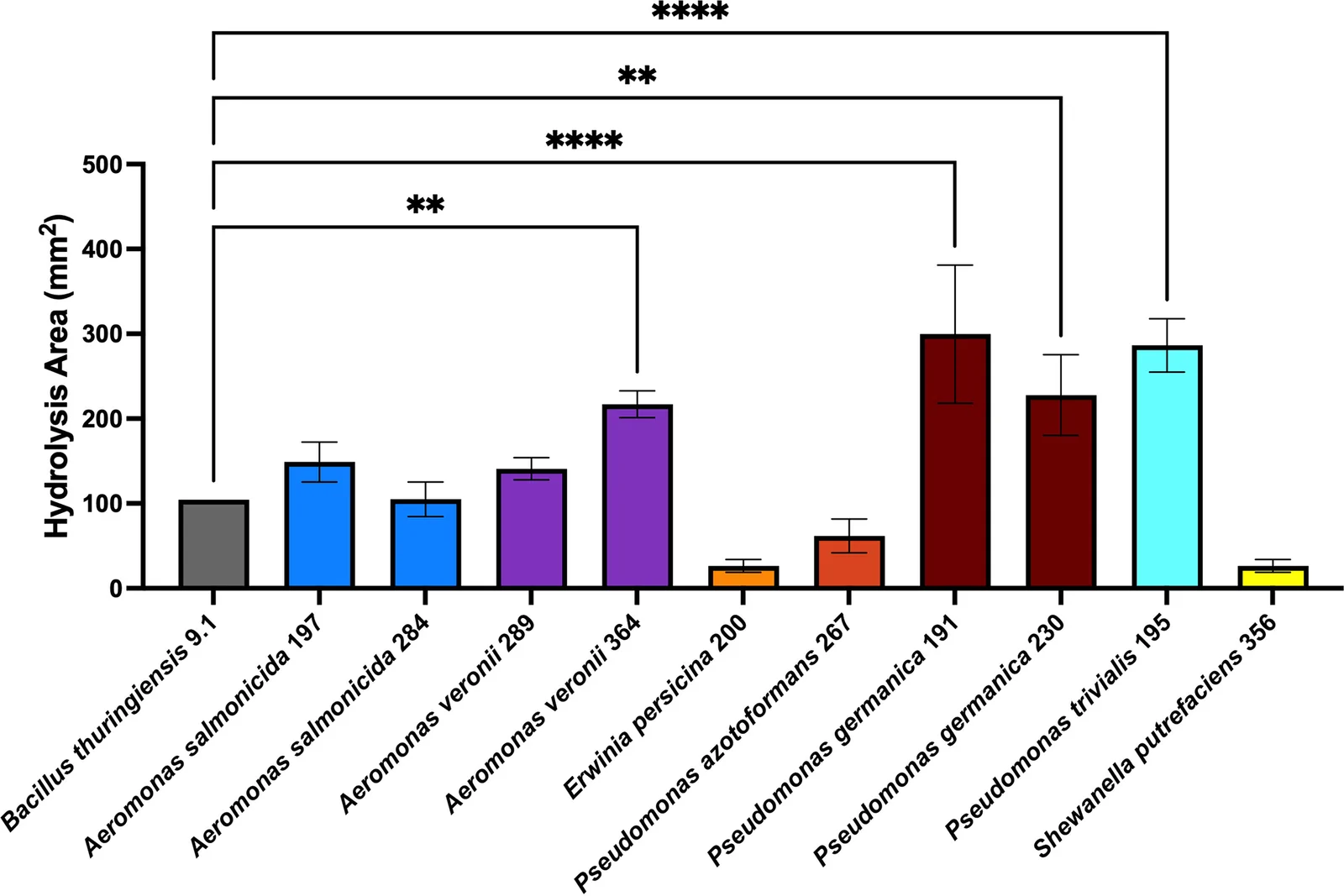
Esterase activity of stickleback-derived isolates after 48 h on 1% tributyrin agar. Esterase activity was quantified and compared to positive control 9.1 (12). Statistical significance was determined by one-way ANOVA and is indicated by *P* < 0.002 (**) and *P* < 0.0001 (****). Error bars indicate standard deviations.

Among the isolates with the highest esterase activity, 11 of the 21 isolates were *Aeromonas veronii* from Rabbit Slough (Table S2). Similar to the lipase activity, there were other *A. veronii* strains, some isolated from the same population of stickleback, with lower esterase activity, indicating within-species and within-population variation in esterase activity. For example, two *A. veronii* strains, KMM 358 and KMM 364, both from Rabbit Slough, have significant differences in esterase activity (118.6 ± 12.2 mm^2^ and 217.0 ± 15.9 mm^2^, respectively). Other species, such as *P. germanica* strains KMM 191 and KMM 230, had similar esterase activity (299.8 ± 81.4 mm^2^ and 227.77 ± 47.6 mm^2^, respectively), indicating comparable activity among independently isolated strains (Fig. 1).

All isolates with significant esterase activity were also positive for lipase activity. However, lipase activity did not predict esterase activity, as some lipase positive isolates lacked significant esterase activity.

### Biofilm formation varies by plastic type

While it is known that microbial biofilm formation on plastics contributes to adsorption, desorption, and degradation of plastics (62), comparative studies on biofilm formation across different plastic types are limited. We asked whether the ability of the microbes to form biofilms depends on plastic type.

**Fig 2:**
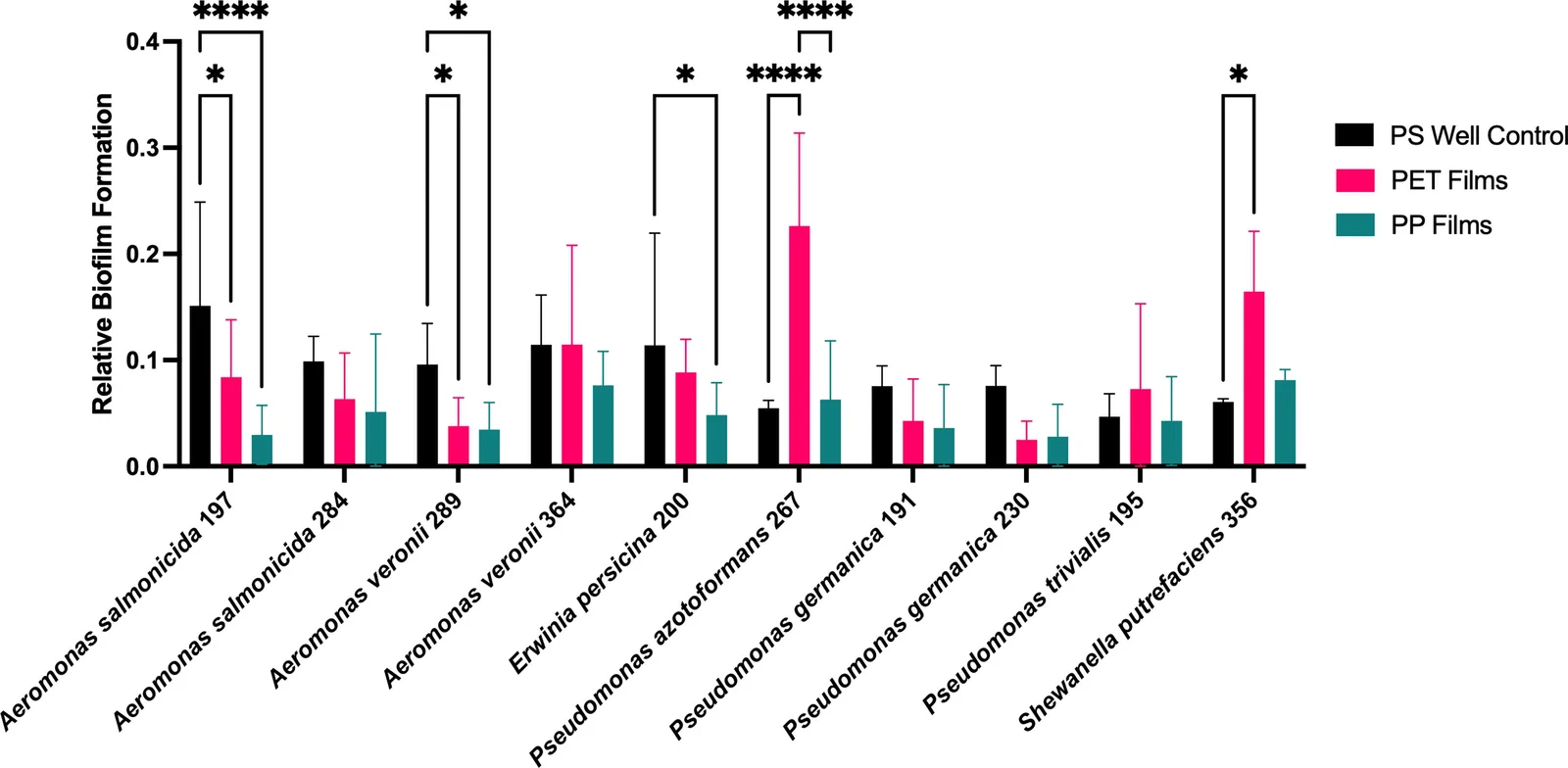
Surface area-normalized relative biofilm formation on PET and PP films compared with a PS-only control well (no added plastic film). Biofilm formation values represent absorbance measurements (OD_590_) normalized to corresponding uninoculated blank controls for each condition that underwent the same crystal violet staining and destaining process. Blank controls consisted of uninoculated wells containing the same substrate (PET film, PP film, or PS-only control well). Relative biofilm formation was corrected using the PS-only control well and normalized to the total surface area. The PS-only control represents the biofilm formation on the PS well surface in the absence of an added PET or PP film. Statistical significance was determined by two-way ANOVA, followed by Tukey’s multiple comparison test, and is represented as *P* < 0.05 (*) and *P* < 0.001 (****). Error bars indicate standard deviations.

Among the 61 biofilm-forming isolates, biofilm formation was generally greater on the PS well surfaces than on the PET or PP films, although this trend was not statistically significant for all isolates. Two isolates, KMM 267 (*Pseudomonas azotoformans*) and KMM 356 (*Shewanella putrefaciens*), exhibited greater RBF on PET films (0.23 ± 0.09 and 0.17 ± 0.06 RBFs, respectively) than on the PS control well, where both isolates exhibited an RBF of 0.06 ± 0.0 on the PS well (Fig. 2).

**Fig 3:**
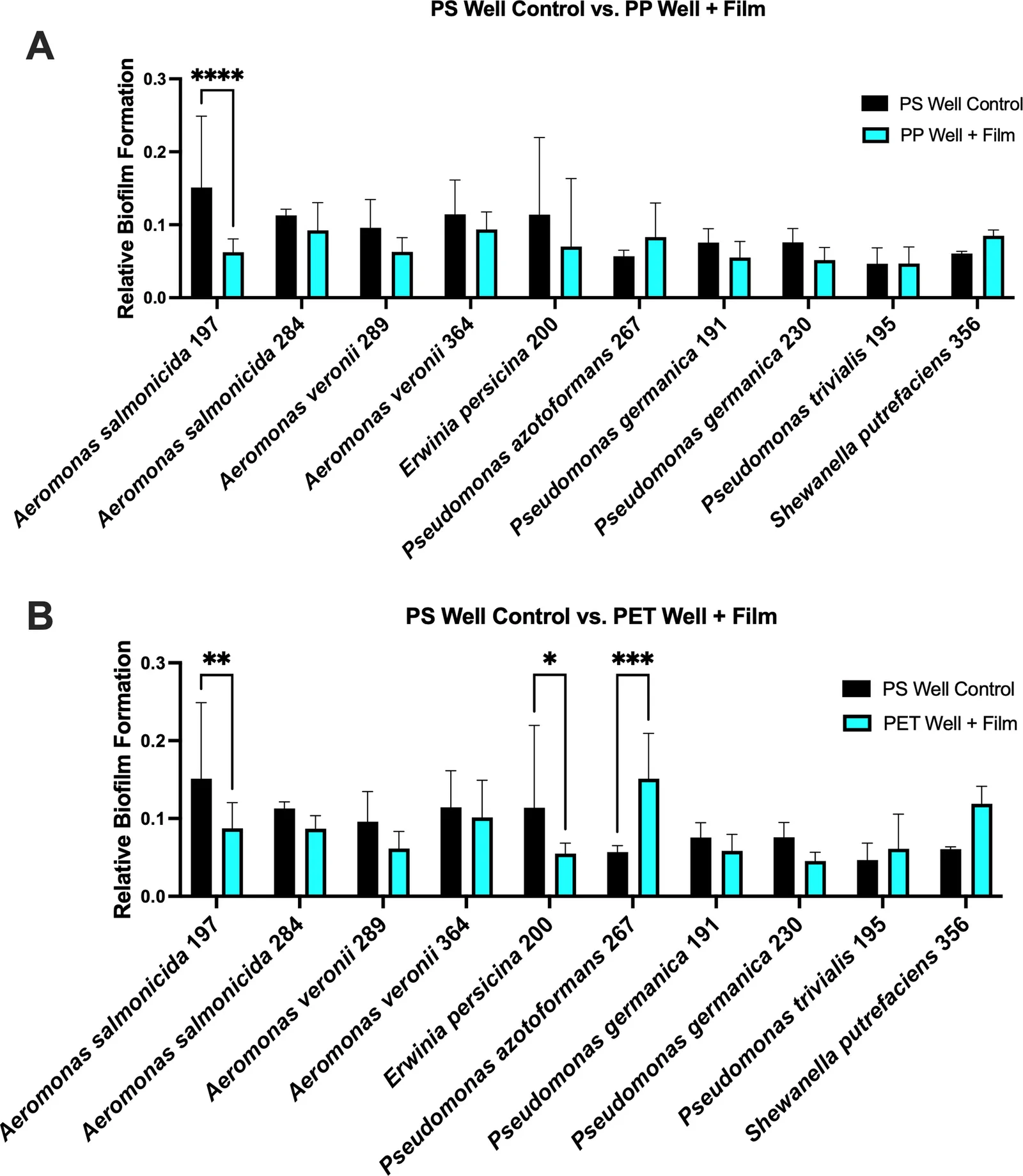
Total biofilm formation in the presence of PP (**A**) and PET films (**B**) compared to a PS-only control well lacking an added plastic film. Total biofilm formation was calculated as the combined biofilm present on the plastic film surface and the PS well surface. Relative biofilm formation values were compared to PS-only control wells containing no added PET or PP film. Statistical significance was determined using two-way ANOVA followed by Tukey’s multiple comparison test and is represented as *P* < 0.05 (*), *P* < 0.01 (**), *P* < 0.001 (***), and *P* < 0.0001 (****). Error bars indicate standard deviations.

We next asked whether the presence of PET or PP influenced total biofilm formation relative to the PS-only control wells without an added plastic film. This analysis quantified total biofilm formation by combining biofilm formed on both the plastic film and the PS well surface and comparing it to biofilm formed in PS-only control wells. Some isolates, such as KMM 267 (*P. azotoformans*), formed more total biofilm in the presence of PET (0.15 ± 0.06 RBF) than in the PS-only control well (0.06 ± 0.01 RBF). Conversely, KMM 200 (*Erwinia persicina*) and KMM 197 (*Aeromonas salmonicida*) showed reduced total biofilm in the presence of PET or PP compared to the PS-only control (Fig. 3A and B). Similarly, other isolates such as KMM 244 (*Buttiauxella ferragutiae*) and KMM 255 (*Lysinibacillus sphaericus*) showed reduced biofilm formation with plastic compared to the PS-only control (Table S1). KMM 222 (*Pseudomonas peli*) formed more biofilm in the presence of PP (0.15 ± 0.1 RBF) compared to the control (0.05 ± 0.02 RBF); however, there was no significant difference observed between the control and the well with PET added (Table S1).

### Surface characterization of polypropylene reveals isolate-specific oxidation

**Fig 4:**
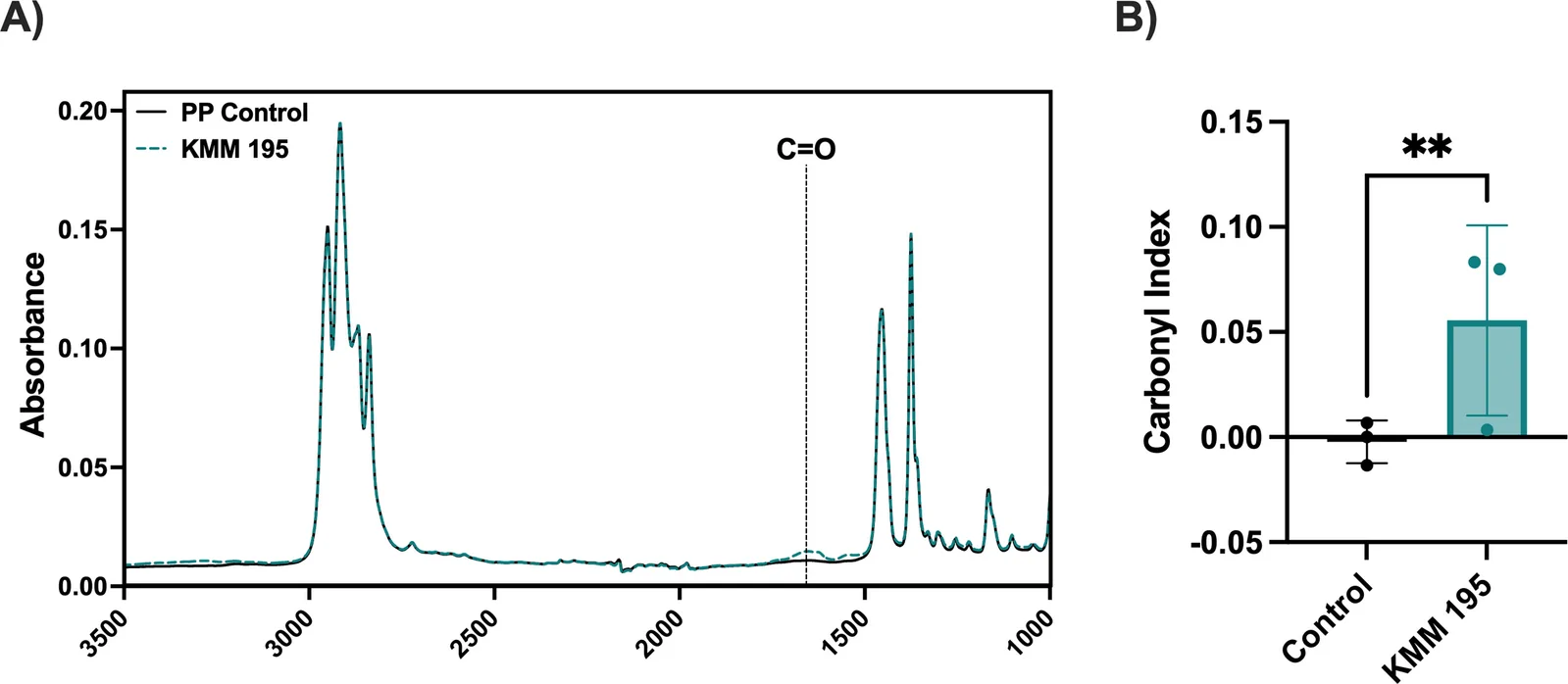
ATR-FTIR analysis shows increased oxidation in PP films incubated with KMM 195. (**A**) Averaged ATR-FTIR of PP films from uninoculated control (black) and films incubated with KMM 195 (teal). Spectra represent the mean of three plastic films, each analyzed by three ATR-FTIR scans (*n* = 9 total spectra per treatment). (**B**) Carbonyl index of PP films incubated without microbes or with KMM 195. Each point represents an individual plastic film, and carbonyl index values were calculated from the mean of three ATR-FTIR scans collected from each film. Bars indicate means ± standard deviations. Statistical significance was determined using two-way ANOVA followed by Dunnett’s multiple comparison test and is represented as *P* < 0.001 (**).

ATR-FTIR was performed on 54 microbes on both PET and PP films. ATR-FTIR analysis revealed no evidence of PET surface oxidation. Evidence of polymer surface oxidation was only observed on the PP films inoculated with KMM 195 (*P. trivialis*), indicated by the formation of a carbonyl stretch in the spectra (Fig. 4A). Carbonyl index calculations (57) confirmed that PP incubated with KMM 195 exhibited a significantly higher carbonyl index compared to the negative control (Fig. 4B), supporting the potential for surface oxidation and chemical modification. Notably, KMM 195 was the only *P. trivialis* isolate out of the tested isolates and was obtained from Westchester Lagoon, a population with high human activity, which may have contributed to its increased plastic-degrading potential (14).

### Co-culture interactions variably alter lipase and esterase activity

**Fig 5:**
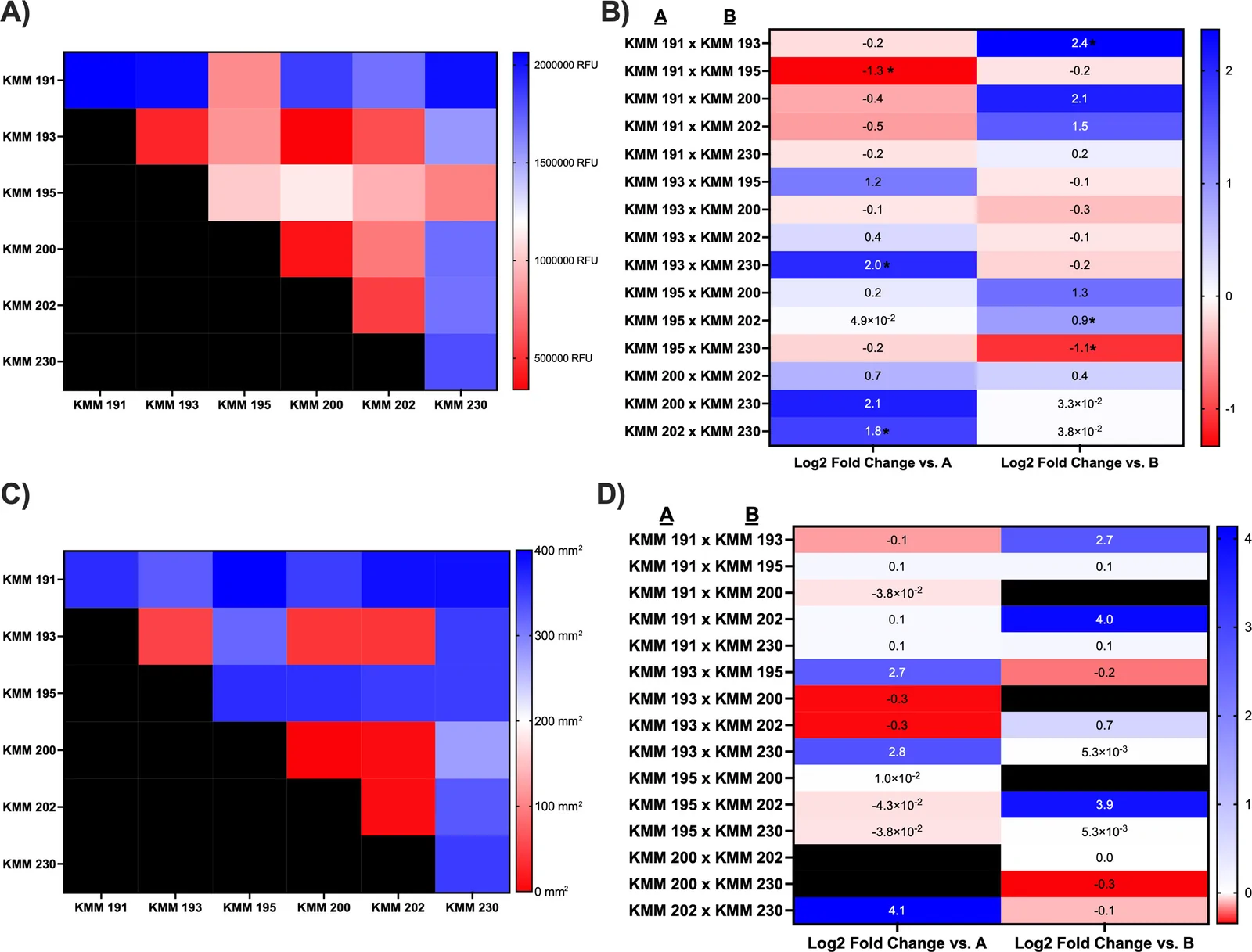
Lipase and esterase activity and fold change analysis in monoculture and co-culture. (**A**) Heat map of lipase activity (reported as RFU) for each monoculture and co-culture. Black cells are repeated comparisons. Exact lipase activity values for all monocultures and co-culture combinations are provided in Table S4. (**B**) Log_2_ fold change in lipase activity for each co-culture relative to monoculture partners A (left) and microbe B (right). Positive values indicate increased lipase activity in co-culture, and negative values indicate decreased activity. Statistical significance was determined by a Wilcoxon signed-rank test relative to a null expectation of zero fold change and is indicated by *P* < 0.05 (*). (**C**) Heat map showing esterase activity (reported as square millimeter hydrolysis area) for each monoculture and co-culture combination. Exact esterase activity values for all monocultures and co-culture combinations are provided in Table S5. (**D**) Log_2_ fold change in esterase activity for each co-culture relative to monoculture partners A (left) and B (right). Positive values indicate increased esterase activity, and negative values indicate decreased activity. Lipase and esterase activities represent the mean of technical replicates for each condition. Black cells represent repeated or non-applicable comparisons.

We evaluated lipase and esterase activity in select co-cultures and compared them to the corresponding monocultures. Lipase activity in co-culture was highly variable, with both enhancement and suppression observed, depending on the strain combination. For example, the co-cultures containing KMM 230 (*P. germanica*, 1.8 × 10^6^ ± 7.9 × 10^5^ RFU) showed significant increases in lipase activity relative to KMM 193 (*Pseudomonas fragi*, log_2_ fold change = 2.0, *P* = 0.031) and KMM 202 (*Pantoea agglomerans*, log_2_ fold change = 1.8, *P* = 0.031) (Fig. 5A and B; Table S3 and S4).

In contrast, the co-culture of KMM 191 (*P. germanica*, 2.1 × 10^6^ ± 4.0 × 10^5^ RFU) and KMM 195 (1.0 × 10^6^ ± 5.4 × 10^5^ RFU) resulted in a significant decrease in lipase activity relative to KMM 191 (log_2_ fold change = −1.3, *P* = 0.031). Similarly, the co-culture of KMM 195 (1.0 × 10^6^ ± 5.4 × 10^5^ RFU) and KMM 230 (*P. germanica*, 1.8 × 10^6^ ± 7.9 × 10^5^ RFU) resulted in a decrease in lipase activity relative to KMM 230 (log_2_ fold change = −1.1, *P* = 0.031). Other co-culture combinations exhibited positive or negative fold changes but did not differ significantly (Fig. 5A and B; Table S3 and S4). Together, these results indicate that lipase activity in co-culture is highly variable and strain dependent, with specific interactions leading to either enhancement or suppression relative to monoculture activity.

Co-cultures were also evaluated for esterase activity and compared to corresponding monocultures (Fig. 5C and D; Table S5). Although several co-culture combinations exhibited positive or negative log_2_ fold changes relative to one of the monoculture partners, none of these differences were significant when assessed using a Wilcoxon signed-rank test. This suggests that while some co-cultures exhibit enhanced esterase activity relative to a single partner, the overall activity of the co-cultures does not significantly enhance or inhibit the activity of both contributing monocultures or the esterase activity is due to one strain in the co-culture.

### Co-culture interactions modulate biofilm formation on PET and PP films in glass-tube incubations

**Fig 6:**
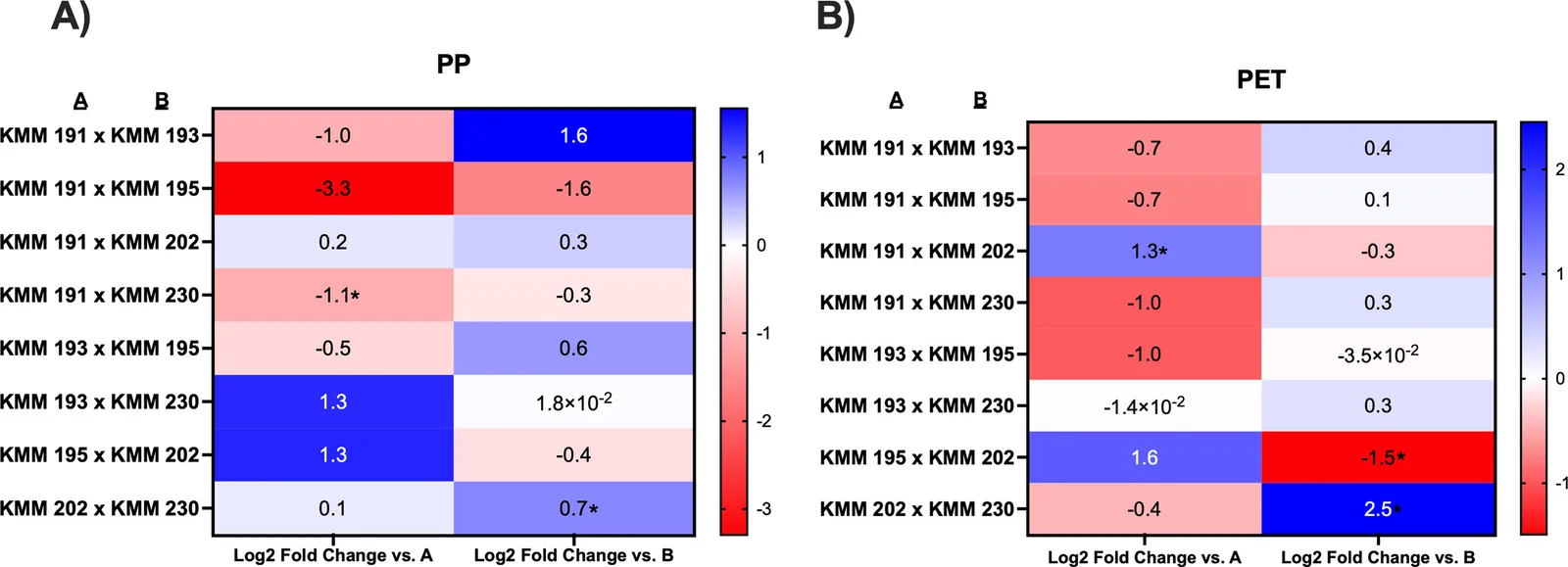
Log_2_ fold change in relative biofilm formation on PP (**A**) and PET (**B**) for each co-culture relative to monoculture partners A (left) and B (right). Positive values (blue) indicate increased biofilm formation in co-culture, and negative values (red) indicate decreased biofilm formation. Relative biofilm formation values represent the mean of technical replicates for each condition. Statistical significance was determined by a Wilcoxon signed-rank test relative to a null expectation of zero-fold change and is indicated by *P* values <0.05 (*).

Relative biofilm formation was compared between monocultures and co-cultures on both PP and PET films. Notably, the co-culture between KMM 191 and KMM 195 showed significantly inhibited biofilm formation on PP when in co-culture compared to monoculture, with a −3.3 log_2_ fold decrease compared to KMM 191 and a −1.6 log_2_ fold decrease compared to KMM 195 (Fig. 6A). Interestingly, the co-culture of KMM 191 and KMM 195 showed different results on PET, where there was a −0.7 log_2_ fold decrease compared to KMM 191 and a 0.1 log_2_ fold increase over KMM 195 (Fig. 6B), which could suggest that biofilm formation is influenced by not only microbial interactions but also the type of plastic surface.

### Co-culture of KMM 191 (*P. germanica*) suppresses PP surface oxidation of KMM 195 (*P. trivialis*)

**Fig 7:**
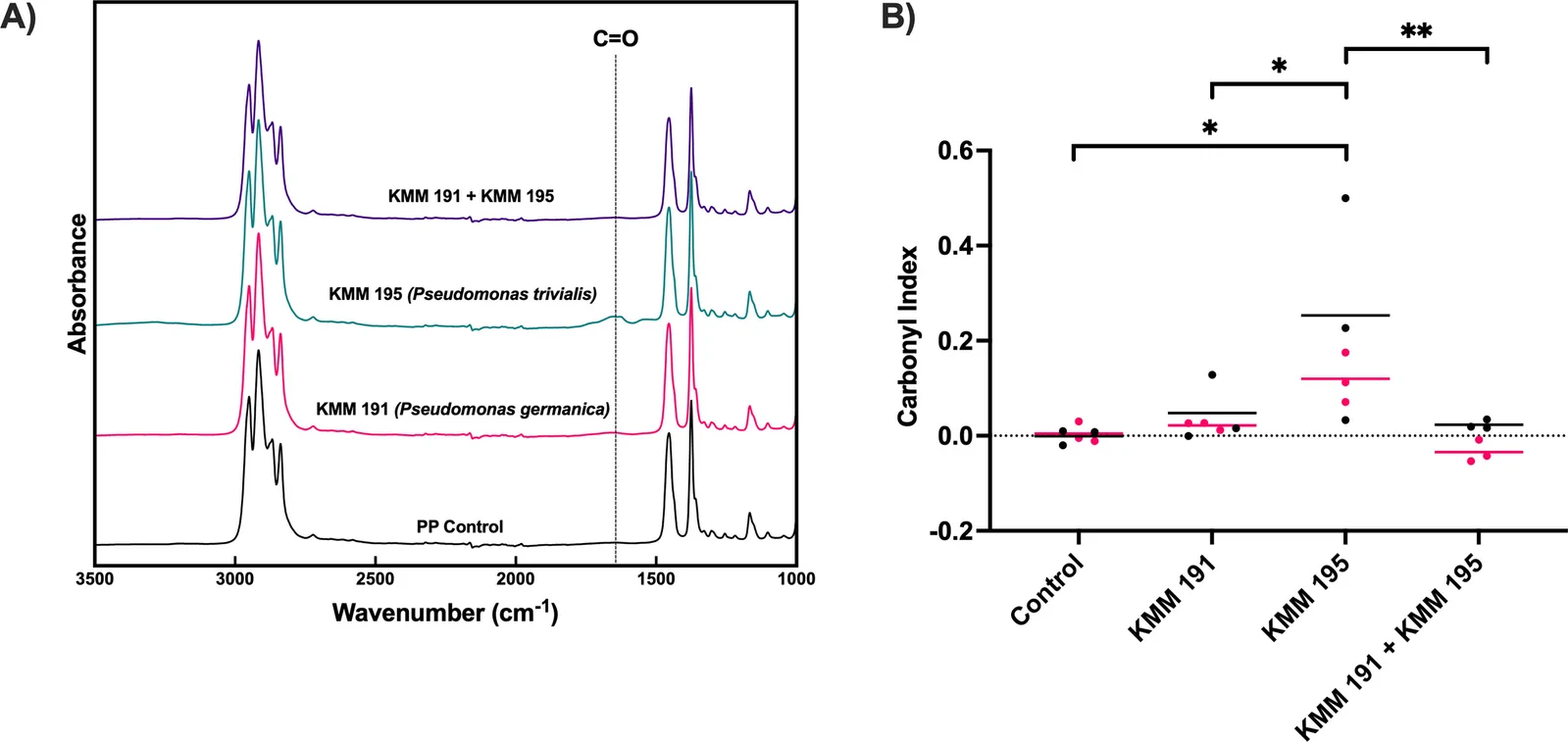
ATR-FTIR analysis reveals increased surface oxidation of PP films incubated with KMM 195, whereas co-culture with KMM 191 suppressed oxidative changes. (**A**) Averaged ATR-FTIR spectra across biological replicates of PP films from the uninoculated control (black), KMM 191 (pink), KMM 195 (teal), and the KMM 191 + KMM 195 co-culture (purple). Spectra represent two biological replicates per treatment, each consisting of three independent PP films analyzed by three ATR-FTIR scans per film (*n* = 27 total spectra per treatment). A dotted line highlights the emerging carbonyl peak in the KMM 195 monoculture. Inoculated samples were also compared to an untreated blank (not shown), which produced a spectrum identical to the uninoculated control. (**B**) Carbonyl index of PP films inoculated with KMM 191 and KMM 195 as monocultures or as a co-culture. Each point represents an individual PP film, and carbonyl index values were calculated from the mean of three ATR-FTIR scans of each film, with colors distinguishing biological replicates. Lines represent the mean for each biological replicate. Statistical significance was first assessed within each biological replicate, with only the KMM 195 treatment group significantly different from the control. The statistical significance reflects the combined analysis across both biological replicates and was determined by two-way ANOVA followed by Tukey’s multiple comparison test and is represented as *P* < 0.05 (*), *P* < 0.01 (**).

Co-cultures for ATR-FTIR analysis were selected to test whether KMM 195 (*P. trivialis*) oxidation would be enhanced or suppressed. Co-cultures of KMM 195 with KMM 191 (*P. germanica*) (Fig. 7B), KMM 193 (*P. fragi*), and KMM 230 (*P. germanica*) (data not shown) reduced the carbonyl index, indicating the suppression of plastic-degrading potential in KMM 195. Since KMM 191 had higher lipase and esterase activity than KMM 193 and belonged to the same species as KMM 230, subsequent analyses focused on interactions between KMM 191 and KMM 195.

**Fig 8:**
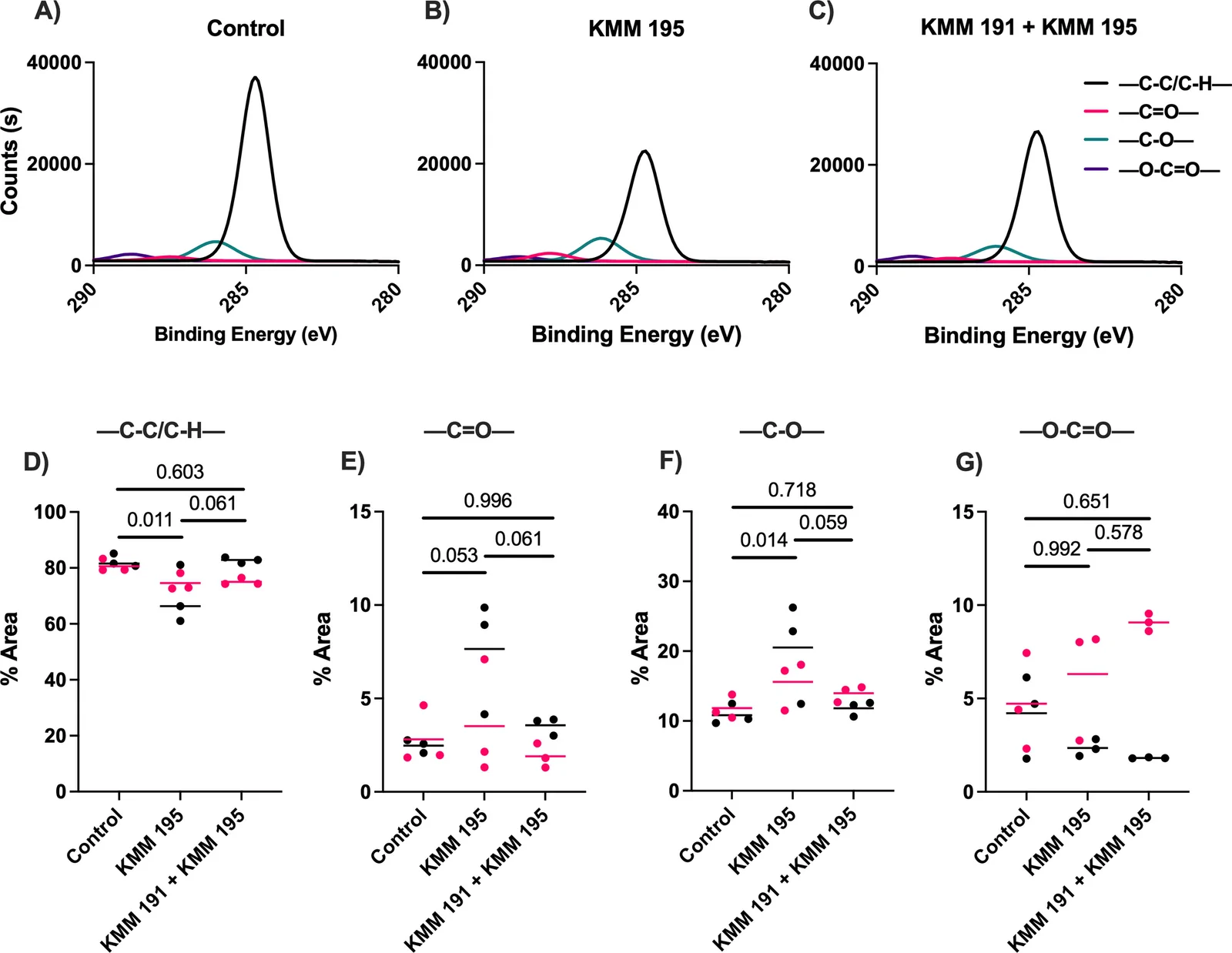
XPS analysis shows that KMM 195 induces oxidative surface modification of PP, while this effect is reduced in co-culture with KMM 191. (**A–C**) High-resolution XPS C 1s spectra of PP films from the uninoculated control, KMM 195, and the KMM 191 + KMM 195 co-culture. (**D–G**) Relative peak area percentage of C-C/C-H, C=O, C-O, and O-C=O groups from C 1s peak deconvolution. Each point represents an individual plastic film, calculated by the mean of three XPS scans of each film, with colors distinguishing biological replicates. Horizontal lines represent the mean for each biological replicate. *P* values shown above the brackets indicate pairwise comparisons between treatment groups determined using two-way ANOVA followed by Tukey’s multiple comparison test.

XPS analysis of PP films incubated with KMM 195 (Fig. 8A and B) showed a decrease in the C-C/C-H peak at 284.9 eV and a corresponding increase in oxidized carbon species relative to the negative control. Peak area quantification (Fig. 8D through G), analyzed using two-way ANOVA with Tukey’s *post hoc* test, confirmed a significant reduction in C-C/C-H bonds (*P* = 0.008) and an increase in C-O bonds (286.7 eV, *P* = 0.01), while C=O (287.9 eV) peak area increased and approached significance (*P* = 0.053), supporting PP surface oxidation under monoculture conditions. In contrast, PP films incubated with KMM 191 and KMM 195 showed no significant differences from the control (Fig. 8D through F), indicating that KMM 191 suppressed the degradative activity of KMM 195. Comparisons of C-C/C-H, C-O, and C=O peak areas between KMM 195 and the KMM 191 + KMM 195 co-culture trended toward *P* = 0.061, 0.061, and 0.059, respectively, indicating attenuation of oxidation in co-culture.

PERMANOVA revealed that the KMM 195 treatment group differed significantly from the control (*F* = 4.71, *R*^2^ = 0.320, *P* = 0.039). In contrast, no significant differences were detected between the control and the KMM 191 + KMM 195 treatment group (*F* = 0.426, *R*^2^ = 0.041, *P* = 0.604), suggesting that co-culture conditions mitigate these changes in the surface chemistry. Direct comparisons of KMM 195 to the KMM 191 + KMM 195 treatment group were not significant (*P* = 0.127) but exhibited a moderate effect size (*F* = 2.54, *R*^2^ = 0.203), suggesting the chemical changes observed in monoculture are attenuated when KMM 195 is co-cultured with KMM 191.

**Fig 9:**
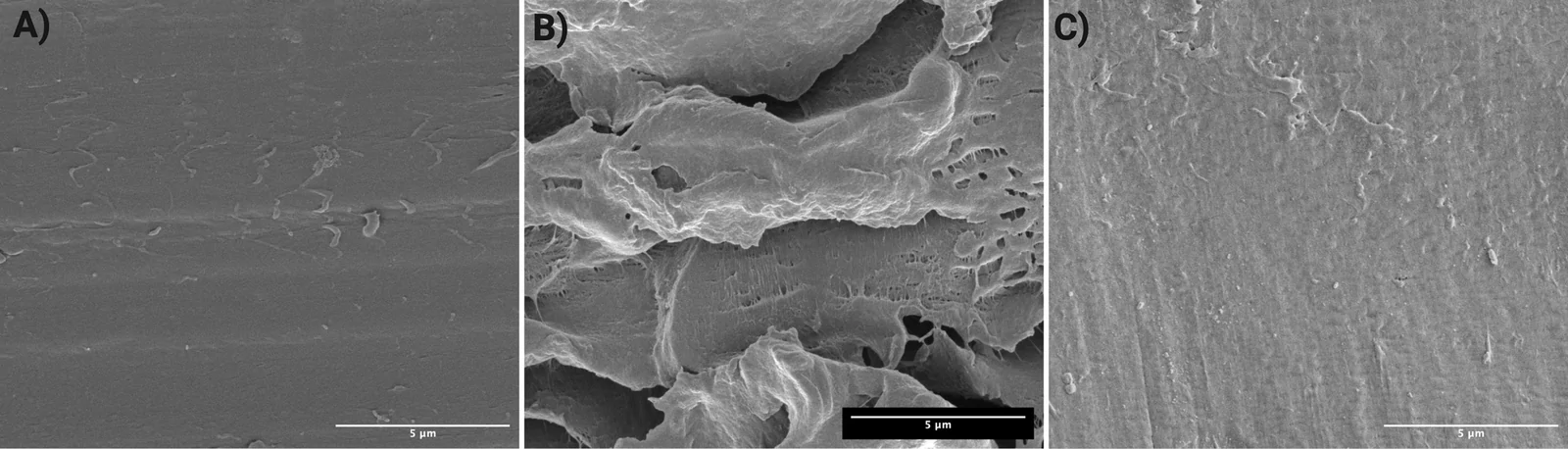
SEM images of PP film cross-sections. (**A**) Uninoculated control showing a smooth and intact surface. (**B**) PP film incubated with KMM 195 displaying increased surface roughness after 8 weeks of incubation in MSM. (**C**) PP film incubated with KMM 191 and KMM 195 co-culture displaying decreased surface roughness after 8 weeks of incubation in MSM.

SEM revealed increased surface roughness and structural disruption in the cross-section of the films incubated with KMM 195 (Fig. 9B) compared to the intact surface of the uninoculated control (Fig. 9A), which is consistent with the PP oxidation. In contrast, the PP film incubated with the KMM 191 and KMM 195 co-culture revealed decreased surface roughness with a surface texture similar to that of the control (Fig. 9C). Overall, these morphological changes are consistent with the chemical changes shown through ATR-FTIR and XPS, further indicating that incubation with KMM 195 promotes PP surface oxidation and modification under monoculture conditions. These surface-level observations parallel the enhanced lipase activity and biofilm formation observed for KMM 195 in monoculture, and the suppression of these activities in co-culture, linking enzymatic function and surface colonization dynamics to the resulting surface modification phenotype.

## DISCUSSION

In this study, we explored the enzymatic activity, biofilm formation, and degradation potential of 184 bacterial isolates from the guts of threespine stickleback collected across six cold-water locations in Alaska. Lipase activity, esterase activity, and biofilm formation were used as screening-level indicators of plastic-degradation potential; however, our findings show that these assays alone did not reliably predict polymer surface modification. While many isolates exhibited biofilm formation and hydrolytic enzyme activity, only one isolate (KMM 195, *P. trivialis*) exhibited clear evidence of polypropylene surface modification, highlighting the importance of distinguishing between screening-level indicators and evidence of polymer modification. Notably, no detectable PET surface oxidation was observed under the conditions tested. While many *Pseudomonas* species have been documented to degrade plastics (12, 18, 26, 63), *P. trivialis* has not been previously associated with polymer surface oxidation or modification (64), highlighting a novel capability in this species and extending its functional potential from a cold-water environment where no plastic degraders have been identified. This underscores the importance of examining understudied taxa from non-traditional environments. These findings suggest that plastic-degrading activities are unevenly distributed across gut-associated microbes and that individual strains with enhanced capabilities may contribute to the response of the gut microbiome to plastic exposure. Although fish sampling was uneven and thus not analyzed, the majority of isolates exhibiting hydrolytic enzyme activity and biofilm formation were isolated from fish living in lakes with moderate to high human activity, suggesting human activity could contribute to the plastic-degrading potential of a microbiome, although this relationship was not directly tested and warrants further investigation.

Beyond monoculture experiments, co-culture assays revealed that microbial interactions can significantly influence plastic-degradation potential. Co-cultures with KMM 195 exhibited suppressed lipase activity and biofilm formation on PP when co-cultured with KMM 191 and KMM 230 (*P. germanica*). Because these three strains were isolated from the same stickleback population (Table S1), these results emphasize that members of the same microbiota could directly suppress the degradation potential of one another, and KMM 195 may possess intrinsic plastic-degrading capabilities. This inhibition may arise through resource competition, quorum sensing interference, or secretion of inhibitory enzymes, all of which have been observed in previous studies (65–68). It is also possible that the introduction of another microbial species could counteract this suppression either by inhibiting the inhibitory strain or by restoring degradative activity through cross-feeding or signaling interactions (69, 70). Together, these results reinforce the need to evaluate plastic degradation within the context of microbial consortia, rather than relying exclusively on monoculture-based assessments, as degradation rates are likely shaped by complex networks of cooperation and inhibition (71, 72). This consideration is particularly important in cold-water environments, where lower temperatures are expected to slow chemical weathering and enzymatic reactions (73–75), contributing to prolonged persistence of environmental plastics.

The identification of a cold-water *P. trivialis* strain with plastic-degrading capabilities brings potential for biotechnological applications. This observation expands the metabolic versatility of the species originally isolated in the phyllosphere in a warmer climate (64) and suggests that this cold-adapted species could possess a group of enzymes with plastic-degrading potential in low-temperature environments. Psychrophilic enzymes with plastic-degrading activity could offer advantages for bioremediation under ambient temperatures where thermophilic enzymes are less effective. Moreover, advances in enzyme engineering have demonstrated the potential of increasing hydrolase activity for polymer degradation (21, 30–32, 76), highlighting the potential for psychrophilic enzymes as candidates for biotechnological advances.

While our assays provided low-cost, scalable methods to assess plastic-degradation potential through enzymatic assays and biofilm formation on plastic films, they do not identify specific enzymes or metabolic pathways responsible for degradation. Furthermore, these assays should be interpreted as screening-level indicators of plastic-degradation potential rather than direct evidence of polymer degradation. Few hydrolases have been linked to specific plastic-degrading functions (15, 33), and future work will combine whole genome sequencing, transcriptomics, and targeted enzyme assays to identify potential degradation pathways (77). However, gut-associated microbial communities in other organisms have been shown to contribute to plastic degradation; for example, multiple types of insect larvae have gut microbes capable of degrading plastics such as polyethylene and polystyrene (40, 42, 78). In this context, the isolation of microbes from different fish populations provides an opportunity for future studies to assess whether plastic-degrading potential varies with human activity. Moreover, the plastic films used in this study were produced under controlled conditions that lack the additives, dyes, and weathering effects that characterize most environmental plastics (2, 75, 79, 80), and degradation under standard laboratory incubation conditions may differ from activity occurring in natural cold-water systems. Testing gut isolates on weathered plastics with environmental modifications would determine whether the degradation activities observed here reflect ecologically relevant degradation processes. Importantly, the analytical approaches used in this study provide evidence of chemical and morphological modifications to the polypropylene surfaces but do not directly quantify polymer mass loss, release of degradation intermediates, or mineralization. Therefore, our results should be interpreted as evidence of polymer surface oxidation and modification rather than definitive proof of complete biodegradation. Additional approaches such as mass-loss measurements, quantification of released degradation products, and stable isotope probing with (14) C-labeled plastics could confirm the uptake of degradation products into the microbe (81). Mock community experiments that combine isolates with complementary traits (9, 11, 12, 19, 82) would also help determine whether cooperative interactions could minimize the inhibitory interactions observed in co-culture experiments. Ultimately, these directions will clarify whether this degradation potential could be translated into a complex natural ecosystem. By addressing this, future work will help determine whether the gut microbiome of cold-water fish represents a significant source of novel enzymes or communities for bioremediation purposes.

### Conclusion

The threespine stickleback gut microbiome contains bacterial strains with plastic-degrading potential and may contribute to the transformation of plastic compounds in cold-water environments. These findings highlight the potential and complexity of microbiome-driven bioremediation. Individual strains such as KMM 195 can oxidize and modify polypropylene surfaces, but this activity is shaped by intra-community interactions that may suppress this capability. Together, these results contribute to a growing understanding of how host-associated microbes influence interactions with environmental plastics and suggest that microbial modification of plastic surfaces is influenced by microbial community dynamics and points toward the gut microbiome as a potential source for harboring novel cold-adapted plastic-degrading bacteria.

## Data Availability

The data that support the findings of this study are openly available in Zenodo (https://doi.org/10.5281/zenodo.22680089). Stickleback strain identifications have been deposited in GenBank under accession numbers PX470357 and PX519477–PX519659. The graphical abstract is available at BioRender.

## SUPPLEMENTAL MATERIAL

Supplemental tables msystems.00599-26-s0001.docx Tables S1-S5.

ASM does not own the copyrights to Supplemental Material that may be linked to, or accessed through, an article. The authors have granted ASM a non-exclusive, world-wide license to publish the Supplemental Material files. Please contact the corresponding author directly for reuse.
